# Tranexamic acid alters the immunophenotype of phagocytes after lower limb surgery

**DOI:** 10.1186/s12959-022-00373-3

**Published:** 2022-04-11

**Authors:** Dominik F. Draxler, Gryselda Hanafi, Saffanah Zahra, Fiona McCutcheon, Heidi Ho, Charithani B. Keragala, Zikou Liu, David Daly, Thomas Painter, Sophia Wallace, Magdalena Plebanski, Paul S. Myles, Robert L. Medcalf

**Affiliations:** 1 Australian Centre for Blood Diseases, Monash University, Melbourne, Australia; 2 Department of Cardiology, University hospital of Bern, Bern, Switzerland; 3 Bern Center for Precision Medicine, Bern, Switzerland; 4 Department of Anaesthesiology and Perioperative Medicine, The Alfred Hospital and Monash University, Melbourne, Australia; 5 Clinical Senior Lecturer, Discipline of Acute Care Medicine, University of Adelaide, Adelaide, Australia; 6 Department of Anaesthesia, Royal Adelaide Hospital, Adelaide, Australia; 7 School of Health and Biomedical Sciences, Royal Melbourne Institute of Technology University, Melbourne, Australia; 8 Vaccine and Infectious Diseases Laboratory, Department of Immunology and Pathology, Monash University, Melbourne, Australia

## Abstract

**Background:**

Tranexamic acid (TXA) is an antifibrinolytic agent frequently used in elective surgery to reduce blood loss. We recently found it also acts as a potent immune-modulator in patients undergoing cardiac surgery.

**Methods:**

Patients undergoing lower limb surgery were enrolled into the “Tranexamic Acid in Lower Limb Arthroplasty” (TALLAS) pilot study. The cellular immune response was characterised longitudinally pre- and post-operatively using full blood examination (FBE) and comprehensive immune cell phenotyping by flowcytometry. Red blood cells and platelets were determined in the FBE and levels of T cell cytokines and the plasmin-antiplasmin complex determined using ELISA.

**Results:**

TXA administration increased the proportion of circulating CD141+ conventional dendritic cells (cDC) on post-operative day (POD) 3. It also reduced the expression of CD83 and TNFR2 on classical monocytes and levels of circulating IL-10 at the end of surgery (EOS) time point, whilst increasing the expression of CCR4 on natural killer (NK) cells at EOS, and reducing TNFR2 on POD-3 on NK cells. Red blood cells and platelets were decreased to a lower extent at POD-1 in the TXA group, representing reduced blood loss.

**Conclusion:**

In this investigation we have extended our examination on the immunomodulatory effects of TXA in surgery by also characterising the end of surgery time point and including B cells and neutrophils in our immune analysis, elucidating new immunophenotypic changes in phagocytes as well as NK cells. This study enhances our understanding of TXA-mediated effects on the haemostatic and immune response in surgery, validating changes in important functional immune cell subsets in orthopaedic patients.

**Supplementary Information:**

The online version contains supplementary material available at 10.1186/s12959-022-00373-3.

## Introduction

The fibrinolytic system, particularly its effector protease plasmin, is now recognised as a significant component in modulating the immune response in various conditions including infection, central nervous system injury and surgery [1]. In a recent study on patients undergoing cardiac surgery, randomised to the antifibrinolytic agent tranexamic acid (TXA) [2] we were able to demonstrate that inhibition of the fibrinolytic cascade results in reduced infection rates in non-diabetic patients [3]. TXA reduced infection rates within 30 days of surgery independent of its blood-sparing effect, which was accompanied by alterations in the expression pattern of immune-activating and immunosuppressive markers on antigen presenting cells, representing an activated cellular immune state. This immune-modulatory effect of TXA was further confirmed by immune profiling of healthy volunteers after 1 g of oral TXA [3].

Orthopaedic surgery, particularly hip- and knee replacement surgery, is a procedure associated with profound blood loss. TXA has been used empirically for this indication for many years, yet concerns about its safety with respect to thromboembolic complications have been raised frequently due to the lack of well-powered randomised controlled trials (RCT). Hence, the TALLAS trial was designed to test efficacy and safety of TXA in orthopaedic surgery in an RCT, giving us the opportunity to study the immune-modulating effect of TXA in this particular surgical patient population, similar to what we have done for cardiac surgery patients [4, 5].

In this investigation we used a similar approach as previously harnessed in the ATACAS trial to characterise the cellular immune response in patients undergoing orthopaedic (hip or knee replacement) surgery randomised to TXA, as part of the “Tranexamic Acid in Lower Limb Arthroplasty” (TALLAS) feasibility study. Additional immune parameters and time points in the peri-operative phase were assessed to further elucidate the immunological effects of TXA.

## Methods

### TALLAS feasibility study design and patients

The “Tranexamic Acid in Lower Limb Arthroplasty” (TALLAS) pilot trial was a feasibility study for a larger multi-centre trial to investigate the efficacy and safety of TXA in patients over 45 years of age, undergoing lower-limb arthroplasty [4]. In this double-blind RCT, TXA was administered at a dose of 15 mg/kg and rate of 50 mg/min intravenously at skin incision (for total hip replacement) or just prior to tourniquet release (for total knee replacement) and repeated at 8 and 16 h post-operatively. EDTA- and citrate-collected blood was obtained from 9 patients (placebo *n* = 4, TXA *n* = 5) before the start of surgery (preOP), at the end of surgery (EOS) as well as on post-operative day 1 (POD-1) and 3 (POD-3). The TALLAS pilot trial and this sub-study were both approved by the hospital’s ethics committee (Alfred Hospital Research Ethics Committee) and each participant provided written informed consent. Samples from additional 10 non-randomised patients were collected after study recruitment had ended, who all received TXA equivalent to the randomised patients. Hence, the dose and duration of TXA treatment was identical across all individuals treated with TXA in this study.

### Sample collection and laboratory analysis

The 9 consecutive patients of the study population were analysed for immunological changes in blood and plasma; 4 were given placebo, and 5 were administered TXA. Blood was drawn into a 10-mL K2-EDTA tube and a 2.7 mL citrate vacutainer tube (BD Biosciences) and samples processed. The same procedure was performed for the 10 additional patients who were not randomised, all receiving TXA.

### Full blood examination

Differential white blood cell counts, red blood cells (RBC) and platelets were assessed in citrated whole blood using the Hemavet 950FS analyser (Drew Scientific, USA) or the Cell-Dyn Emerald analysing system (Abbott, USA).

### Flowcytometry

PBMCs were thawed and stained for flowcytometry according to our published protocol [6]. The assessed myeloid and lymphoid cell populations as well as functional markers were selected as they represent key cells/markers involved in inflammation and immune stimulation as well as immunosuppression.

In brief, up to 2 million freshly thawed PBMCs were washed twice and transferred to a 96-well round-bottom plate (Corning, USA). The staining was then performed with two separate antibody panels.

Panel I was used to identify myeloid cell populations and included: CD33-FITC, HLA-DR-APC/Cy7, CD11c-BV421, CD141-APC, CD1c-PECy7, CD14-AF700, CD16-BV510, CD123-BV650, CD83-PE/Dazzle 594, CD86-PerCP/Cy5.5, PD-L1-BV711, CCR4-BV605, CCR7-BV785, TNFR2-PE, and dead-cell-staining Zombie Yellow (all BioLegend, USA). After incubation for 20 min on ice, cells were washed and resuspended in 100 uL of PBS + 2% FBS + 1%PFA for fixation.

Panel II was used to identify lymphoid cell populations and included: CD3-BV510, CD4-BV650, CD8-AF700, CD56-APC/Cy7, CD25-PerCP/Cy5.5, CD95-APC, CD45R0-BV711, LAP-BV421, CTLA4-PE/Cy7, PD-1-FITC, CCR4-BV605, CCR7-BV785, TNFR2-PE, and dead-cell-staining Zombie Yellow (all BioLegend, USA). Cells were incubated for 20 min on ice, followed by a washing step before they were permeabilized in 200 uL of “Fix/Perm buffer” (eBioscience, USA). After incubation for 30–60 min at room temperature cells were washed using “Perm buffer” (eBioscience) before 50 mL of FoxP3-PE/Dazzle594 antibody diluted in Perm buffer was added for intracellular staining of cells. Samples were incubated for 30 min at room temperature, and then washed again with Perm buffer, and finally resuspended in 100 uL of PBS.

Stained samples were analyzed using a 4-laser LSR Fortessa (BD Biosciences) with BD FACSDiva software (BD Biosciences) for data acquisition. Single stain controls for compensation were generated utilizing UltraComp eBeads (eBioscience) and the analysis of acquired samples was performed in FlowJo (FlowJo, LLC, Ashland, CA) data analysis software.

For the analysis, debris was excluded based on size (FSC) and complexity (SSC). For phenotypic identification of myeloid cell populations, we used SSC as well as the markers CD33, CD123, CD14, CD16, HLA-DR, CD11c, CD141, and CD1c. For the evaluation of cell activation, migration, function, and cell viability, the additional markers CD83, CD86, CCR4, CCR7, PD-L1, TNFR2, and Zombie Yellow for live/dead discrimination were used. The gating strategy for myeloid cells is demonstrated in Suppl. Fig. 1.

Lymphocyte populations were identified using SSC in combination with CD3, CD4, CD8, CD56, CD25, and FoxP3. Cell activation, migration, function, and cell viability were determined with CD45R0, CCR4, CCR7, PD-1, CD95, TNFR2, LAP, CTLA4, and Zombie Yellow for live/dead discrimination. Technical issues with the CD3 stain led to a complete loss in signal for this marker, critical for T cell identification, which is why T cells were excluded from the flowcytometric analysis. The gating strategy for NK cells is illustrated in Suppl. Fig. 2.

For the assessment of absolute cell counts we first determined the frequencies of the individual cell populations identified within the analysed PBMC sample. We then used the combined counts of the lymphocyte and monocyte populations from the corresponding full blood examination (FBE) result to calculate the absolute number of circulating cells (please note that the granulocyte populations are lost during the PBMC isolation process and therefore have to be excluded from the analysis).

The expression strength of the described surface markers was assessed and described as the mean fluorescence intensity (MFI) on the individual cell subsets.

B cells were identified through negative gating as HLA-DR+ CD14- CD11c- CD123- cells, as previously described [7]. The gating strategy for B cells as an addition to our previously established protocol is demonstrated in Suppl. Fig. 3.

### Il-6 ELISA

IL-6 levels were evaluated using an elisakit.com IL-6 ELISA Kit (elisakit.com, Australia) according to the manufacturer’s instructions.

### Il-10 ELISA

IL-10 levels were quantified harnessing a BD OptEIA IL-10 ELISA Kit (BD Biosciences, USA) according to the manufacturer’s instructions.

### IFN-y ELISA

The plasma levels of IFN-y were determined using a JOMAR LIFE RESEARCH RAY-ELH-IFNg-2 ELSIA Kit (Jomar Life Research, Australia) according to the manufacturer’s instructions.

### Plasmin-antiplasmin (PAP) ELISA

Plasma PAP complex levels were quantified using a DRG PAP micro ELISA Kit (DRG Instruments, Germany) according to the manufacturer’s instructions.

### Statistics

All statistical analyses were performed within Prism 7, Graphpad software (La Jolla, USA). The data were compared with preOP levels using a repeated measure one-way ANOVA with Dunnett’s multiple comparisons test. For this analysis, patients for which a time point was missing (FBE results for one patient in the placebo group and flowcytometry results for one patient in the TXA group) had to be entirely excluded from the analysis. For comparisons between the placebo and TXA groups at an individual time point, data were normalised and postoperative time points presented as fold change of preOP level. Results were then analysed using a 2-tailed Student t test. Differences were considered statistically significant if *p* < 0.05. The data sets were assessed for outliers with a ROUT test (Q = 1%). A maximum of two outliers was detected for some of the evaluated parameters in which case the participant was entirely removed from the analysis. Normal distribution of data was confirmed using a Shapiro-Wilk test.

## Results

The last 9 patients of the TALLAS pilot trial recruited at the Alfred hospital in Melbourne, Australia were included in this investigation, of which 4 received placebo and 5 TXA (Table 1). Between the two groups there was no significant difference with respect to sex distribution, age and type of surgery (hip- or knee replacement surgery). Relevant to the study, we also assessed diabetes status and infection within 30 days after surgery. None of the 9 randomised patients had diabetes or developed an infection in the post-operative phase.

**Table 1:** Baseline characteristics of randomised and non-randomised patients

**Characteristics (randomised patients)**	**Placebo (*n*** **= 4)**		**TXA (*n*** **= 5)**		***p*** **value (Mann-Whitney** ***U*** **Test)**
	**n**	**%**	**n**	**%**	
**Female sex**	1	25	4	80	0.21
**Hip replacement**	3	75	3	60	> 0.99
**Infection within 30 days**	0	0	0	0	–
**Diabetes mellitus**	0	0	0	0	–
**Age (a, mean** ***±*** **SEM)**	68.8 ***±*** 4.1		61.6 ***±*** 1.2		0.21
**Characteristics (non-randomised patients)**	**All TXA (*n*** **= 10)**				
	**n**		**%**		
**Female sex**	7		70		
**Hip replacement**	7		70		
**Infection within 30 days**	1		10		
**Diabetes mellitus**	1		10		
**Age (a, mean** ***±*** **SEM)**	62.1 ± 3.1				

After the randomised pilot study was completed, we recruited 10 additional patients fulfilling the study criteria of the pilot trial for this sub-study. All of these patients received TXA (equivalent to the study protocol). There was one diabetes patient, and one of these patients developed an infection in the 30-days post-operative phase (Table 1), which however had no obvious impact on the parameters assessed at the evaluated time points. Importantly, for all of the parameters presented here we confirmed that there was no difference between the TXA patients recruited during the randomised phase and the later recruited patients receiving TXA (Suppl. Fig. 4).

**Fig. 1:**
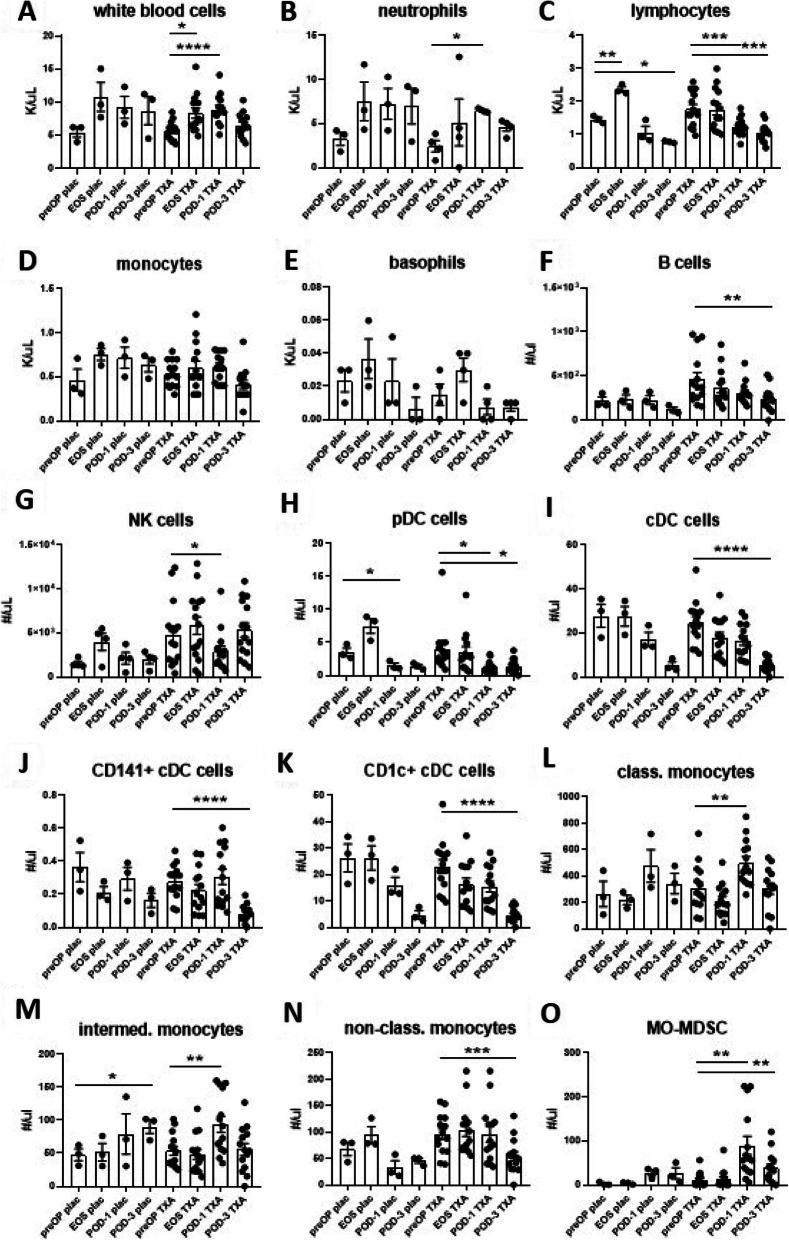
Effects of surgery on white blood cell counts in placebo-and TXA-treated patients. Levels of leukocyte subsets were determined during routine full blood examination (FBE) (**A-E**) and flowcytometry (**F-O**). In placebo-treated patients, an increase in the number of circulating lymphocytes at EOS, followed by a significant reduction at POD-3 (**C**) could be detected. Within the myeloid cell populations we observed a decrease of pDC at POD-1 (**I**) as well as a significant increase of intermediate monocytes at POD-3 (**N**). Counts of the other white blood cell subsets were not significantly altered in this small cohort of placebo-treated patients. In the larger TXA-treated group, an increase in levels of circulating white blood cells overall (**A**), particularly neutrophils (**B**), classical (**L**) and intermediate monocytes (**M**) as well as MO-MDSC was observed, yet a significant decrease in circulating lymphocytes (**C**), particularly in B cells (**F**) and NK cells (**G**), as well as all assessed DC subsets (**H-K**) and non-classical monocytes (**N**) was detected at the various postoperative time points. Data are expressed as mean ± standard error of the mean. Placebo *n* = 3, TXA *n* = 14 (*n* = 4 for neutrophils (**B**) and basophils (**E**)), comparisons with preOP, repeated measures one-way ANOVA with Dunnett’s correction test for multiple comparisons. FBE: full blood examination, preOP: pre-operative, EOS: end of surgery, POD-1: post-operative day 1, POD-3: post-operative day 3, cDC: conventional dendritic cells, pDC: plasmacytoid dendritic cells, MO-MDSC: monocytic myeloid-derived suppressor cells

**Fig. 2:**
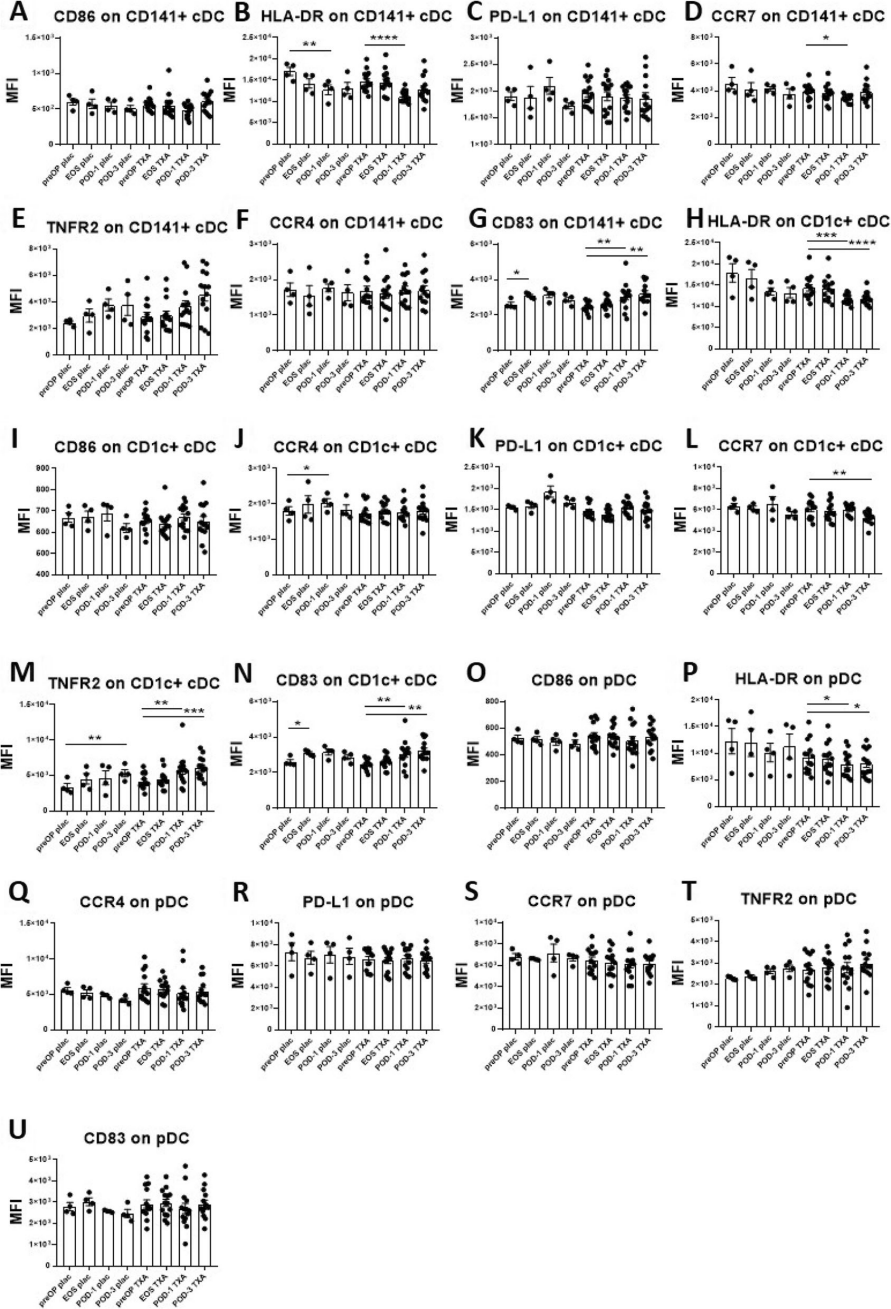
Effects of surgery on DC marker expression in placebo-and TXA-treated patients. The effects of surgery on the expression immune-stimulating and immunosuppressive markers on DC subsets were determined in placebo-treated and TXA-treated patients by flowcytometry. In placebo-treated patients, surgery induced a significant reduction in the expression intensity of HLA-DR on CD141+ cDC at POD-1 (**B**), yet an increase in CD83 at the EOS time point (**G**). In CD1c + cDC’s, an increased expression of CD83 at EOS (**N**), and also of the migration marker CCR4 at POD-1 (**J**), and of TNFR2 on POD-3 (**M**) was detected. In TXA-treated patients, a reduction in the expression of HLA-DR (**B**) as well as CCR7 (**D**), and an increase in CD83 (**G**) on CD141+ cDC could be observed postoperatively. Similarly, in CD1c + cDC attenuated expression of HLA-DR (**H**) and CCR7 (**L**), but an increase of TNFR2 (**M**) and CD83 (**N**) was present. HLA-DR expression was also decreased in pDC in TXA-treated patients (**P**). Data are expressed as mean ± standard error of the mean. Placebo *n* = 4, TXA *n* = 14; comparisons with preOP, repeated measures one-way ANOVA with Dunnett’s correction test for multiple comparisons. preOP: pre-operative, EOS: end of surgery, POD-1: post-operative day 1, POD-3: post-operative day 3, cDC: conventional dendritic cells, pDC: plasmacytoid dendritic cells, monos: monocytes, MFI: mean fluorescence intensity

**Fig. 3:**
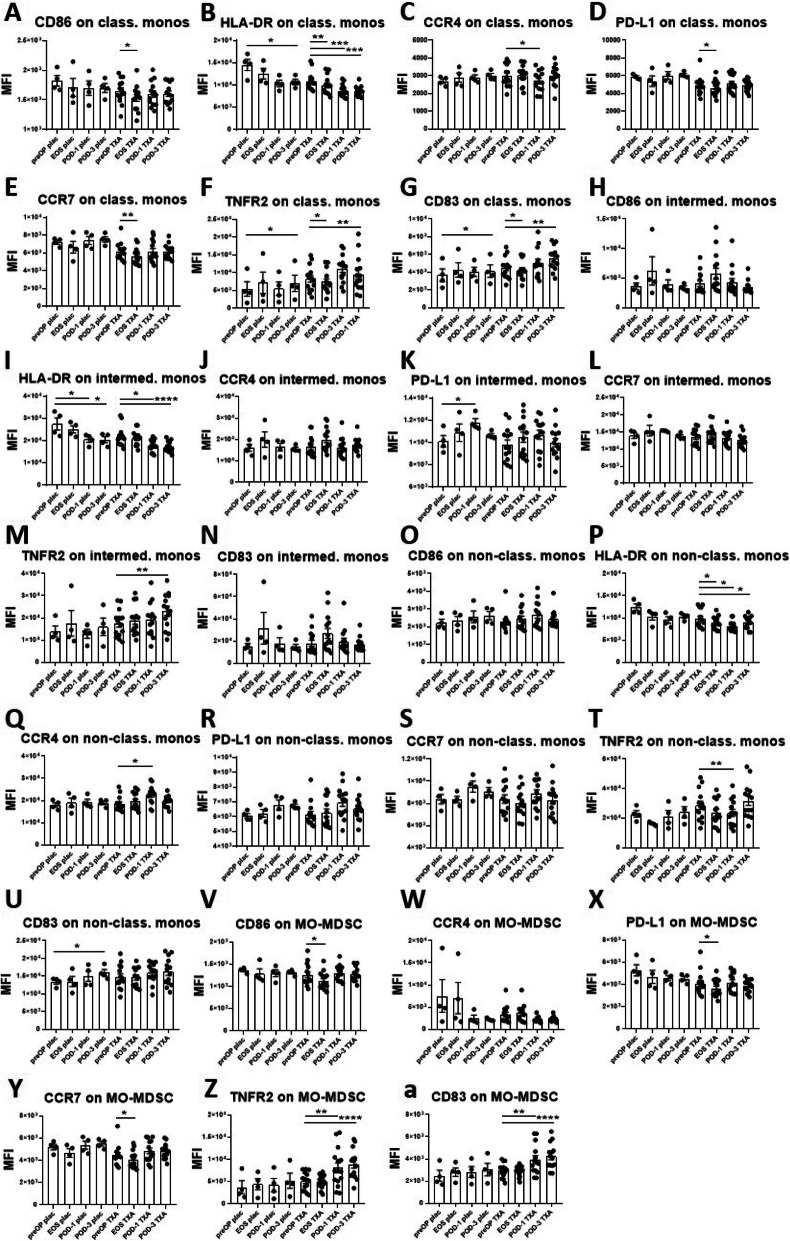
Effects of surgery on monocyte and MO-MDSC marker expression in placebo-and TXA-treated patients. The effects of surgery on the expression immune-stimulating and immunosuppressive markers were evaluated on monocyte subsets and MO-MDSC in placebo-treated and TXA-treated patients using flowcytometry. Classical monocytes displayed a reduction of HLA-DR (**B**), yet an increase of TNFR2 (**F**) and of CD83 (**G**) on POD-3 in placebo-treated patients. A reduction of HLA-DR was also evident on intermediate monocytes on POD-1 and POD-3 (**I**), which in turn showed an increase in PD-L1 expression (**K**). Non-classical monocytes showed enhanced expression of CD83 on POD-3 (**U**). In the TXA group, a postoperative decrease in CD86 (**A**) HLA-DR (**B**), CCR4 (**C**) and PD-L1 (**D**) on classical monocytes could be observed, while an increase in TNFR2 (**F**) and CD83 (**G**) was noticeable. For intermediate monocytes we also obtained comparable results to the placebo group with decreased surface expression of HLA-DR (**I**) and increased TNFR2 (**M**). On non-classical monocytes TXA-treated patients displayed a significant reduction in HLA-DR (**P**) and TNFR2 expression (**T**), yet an upregulation of CCR4 (**Q**). On MO-MDSC CD86 (**V**), PD-L1 (**X**) and CCR7 (**Y**) were downregulated, and TNFR2 (**Z**) and CD83 (**a**) expression enhanced, postoperatively. None of the other parameters was changed postoperatively in the placebo- or TXA treated patients. Data are expressed as mean ± standard error of the mean. Placebo: *n* = 4, TXA *n* = 14. *p = 0.05, **p = 0.01, ***p = 0.001, ****p = 0.0001, comparisons with preOP, repeated measures one-way ANOVA with Dunnett’s correction test for multiple comparisons. preOP: pre-operative, EOS: end of surgery, POD-1: post-operative day 1, POD-3: post-operative day 3, monos: monocytes, MO-MDSC: monocytic myeloid-derived suppressor cells, MFI: mean fluorescence intensity

**Fig. 4:**
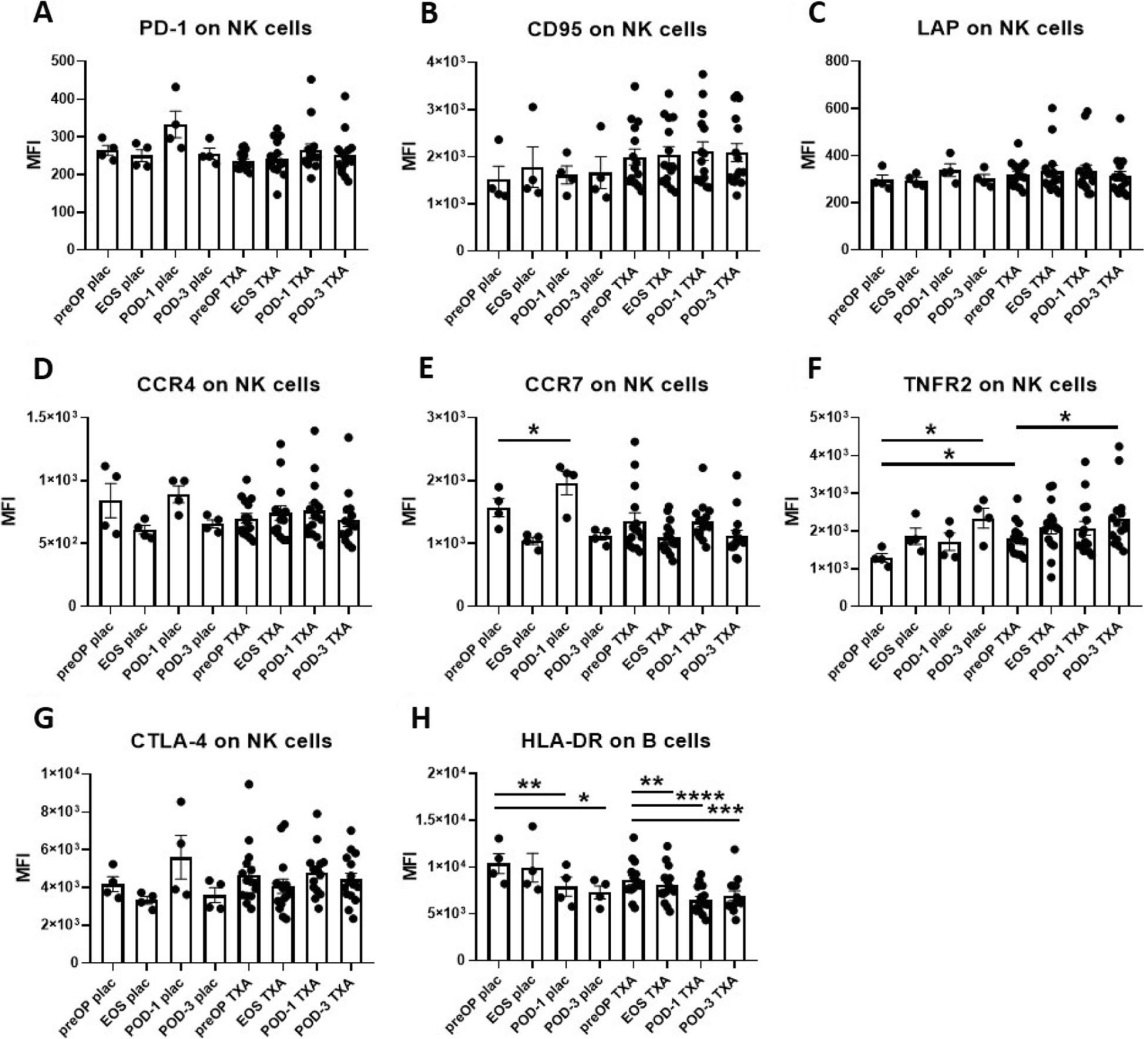
Effects of surgery on NK cell marker expression in placebo-and TXA-treated patients. The effects of surgery on the expression of immune-stimulating and immunosuppressive surface markers were assessed on NK cells (**A-G**) and B cells (**H**) in placebo-treated and TXA-treated patients by flowcytometry. For NK cells, no change was detected at any of the postoperative time points for the expression of PD-1 (**A**), CD95 (**B**), LAP (**C**) and CCR4 (**D**). However, NK cells displayed a reduction of CCR7 at EOS (**E**), and an increase of TNFR2 on POD-3 (**F**). The increase in TNFR2 was also present in the TXA group, which moreover displayed an increase compared to placebo at the preOP time point already (**F**). B cells showed a significant reduction of HLA-DR expression postoperatively in both, placebo- and TXA-treated patients (**H**). Data are expressed as mean ± standard error of the mean. Placebo: *n* = 4, TXA *n* = 15. *p = 0.05, **p = 0.01, ***p = 0.001, ****p = 0.0001, comparisons with preOP, repeated measures one-way ANOVA with Dunnett’s correction test for multiple comparisons. preOP: pre-operative, EOS: end of surgery, POD-1: post-operative day 1, POD-3: post-operative day 3, MFI: mean fluorescence intensity

**Fig. 5:**
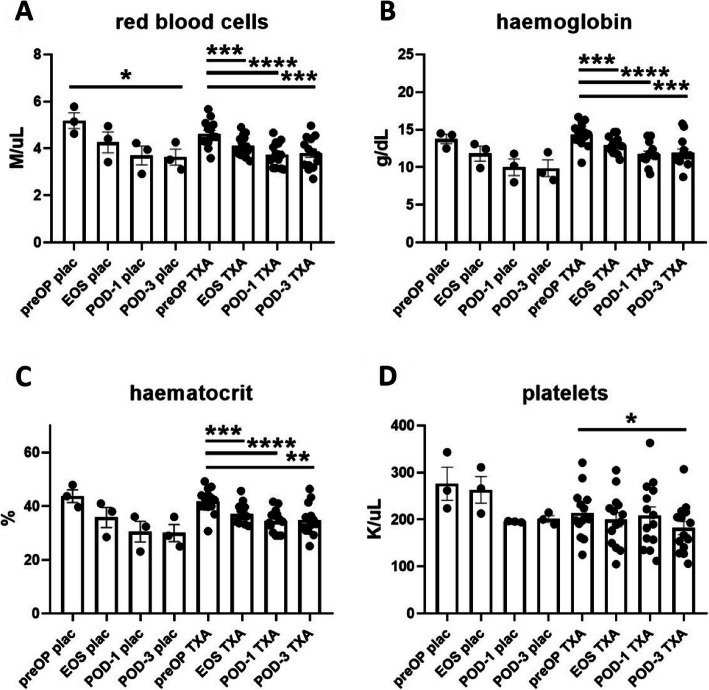
Effects of surgery on the red blood cells and platelets in placebo-and TXA-treated patients. The effects of surgery on levels of red blood cell levels (**A**), haemoglobin (**B**), haematocrit (**C**) and platelets (**D**) in placebo-treated and TXA-treated patients were determined with a haemocytometer. Surgery induced a significant decrease in red blood cell levels at POD-3 (**A**). In the much larger group of TXA-treated patients a significant reduction of red blood cells (**A**), haemoglobin (**B**), and haematocrit (**C**) was evident at all postoperative time points. There was also a significant postsurgical reduction in platelet levels at POD-3 (**D**). Data are expressed as mean ± standard error of the mean. Placebo: *n* = 3, TXA n = 14. *p = 0.05, **p = 0.01, ***p = 0.001, ****p = 0.0001, comparisons of postoperative time points with preOP, repeated measures one-way ANOVA with Dunnett’s correction test for multiple comparisons. FBE: full blood examination, preOP: pre-operative, EOS: end of surgery, POD-1: post-operative day 1, POD-3: post-operative day 3

In the 4 patients receiving placebo we looked at the effects of the surgical procedure alone on immune parameters (Figs. 1, 2, 3 and 4), as well as RBCs and platelets (Fig. 5) directly after surgery (EOS), as well as on POD-1 and POD-3, compared with the preOP time point. We observed an increase in the number of circulating lymphocytes at EOS, followed by a significant reduction at POD-3 (Fig. 1C). Within the myeloid cell populations we observed a decrease of pDC at POD-1 (Fig. 1H) as well as a significant increase of intermediate monocytes at POD-3 (Fig. 1M). None of the other white blood cell subsets assessed with FBE or flowcytometry was significantly changed in count in this small cohort of placebo-treated patients (Fig. 1). In the larger TXA-treated group we observed an increase in levels of circulating white blood cells overall (Fig. 1A), particularly neutrophils (Fig. 1B), classical (Fig. 1L) and intermediate monocytes (Fig. 1M) as well as MO-MDSC, yet a significant decrease in circulating lymphocytes (Fig. 1C), particularly also in B cells (Fig. 1F) and NK cells (Fig. 1G), as well as all assessed DC subsets (Fig. 1H-K) and non-classical monocytes (Fig. 1N) at the various postoperative time points.

Harnessing flowcytometry we also assessed the level of expression of key surface markers involved in cell activation, migration, function, and cell viability on different immune cell subsets, to better characterise potential changes in the functional state of these cells (Figs. 2, 3 and 4). In placebo-treated patients, surgery induced a significant reduction in the expression intensity of HLA-DR on CD141+ cDC at POD-1 (Fig. 2B), yet an increase in CD83 at the EOS time point (Fig. 2G). In CD1c + cDC we also detected an elevated expression of CD83 at EOS (Fig. 2N), of the migration marker CCR4 at POD-1 (Fig. 2J), and of TNFR2 on POD-3 (Fig. 2M). In TXA-treated patients, we also found a reduction in the expression of HLA-DR (Fig. 2B) as well as CCR7 (Fig. 2D), and an increase in CD83 (Fig. 2G) on CD141+ cDC postoperatively. Similarly, in CD1c + cDC we observed attenuated expression of HLA-DR (Fig. 2H) and CCR7 (Fig. 2L), but an increase of TNFR2 (Fig. 2M) and CD83 (Fig. 2N). HLA-DR expression was also decreased in pDC in TXA-treated patients (Fig. 2P).

On classical monocytes we observed a reduction of HLA-DR (Fig. 3B), yet an increase of TNFR2 (Fig. 3F) and of CD83 (Fig. 3G) on POD-3 in placebo-treated patients. A reduction of HLA-DR was also evident on intermediate monocytes on POD-1 and POD-3 (Fig. 3I), which in turn showed an increase in PD-L1 expression (Fig. 3K). On non-classical monocytes CD83 expression was elevated on POD-3 (Fig. 3U). In the TXA group, we observed a postoperative decrease in CD86 (Fig. 3A) HLA-DR (Fig. 3B), CCR4 (Fig. 3C) and PD-L1 (Fig. 3D), yet an increase in TNFR2 (Fig. 3F) and CD83 (Fig. 3G) on classical monocytes. In intermediate monocytes we also obtained comparable results to the placebo group with decreased surface expression of HLA-DR (Fig. 3I) and increased TNFR2 (Fig. 3M). On non-classical monocytes TXA-treated patients displayed a significant reduction in HLA-DR (Fig. 3P) and TNFR2 expression (Fig. 3T), yet an upregulation of CCR4 (Fig. 3Q). On MO-MDSC there was a downregulation of CD86 (Fig. 3V), PD-L1 (Fig. 3X) and CCR7 (Fig. 3Y), and an upregulation of TNFR2 (Fig. 3Z) and CD83 (Fig. 3a) evident, postoperatively.

In NK cells we observed an increase of CCR7 at POD-1 (Fig. 4E) and of TNFR2 on POD-3 (Fig. 4F). The increase in TNFR2 was also present in the TXA group, which surprisingly displayed an increase compared to placebo at the preOP time point (Fig. 4F). B cells displayed a marked reduction of HLA-DR expression postoperatively in both, placebo- and TXA-treated patients (Fig. 4H).

In the 4 individuals receiving placebo, we observed a significant reduction of RBC counts at the POD-3 time point (Fig. 5A). Other parameters associated with the RBC line or platelets were not significantly affected. However, in the much larger group of TXA-treated patients RBC counts, haemoglobin levels and haematocrit were significantly reduced at all postsurgical time points (Fig. 5A-C). In addition, we observed a significant decrease in the levels of platelet at POD-3 in TXA-treated patients (Fig. 5D).

No significant differences were detected postoperatively or between treatment groups at baseline in any of the other data sets shown in Figs. 1, 2, 3, 4 and 5.

**Fig. 6:**
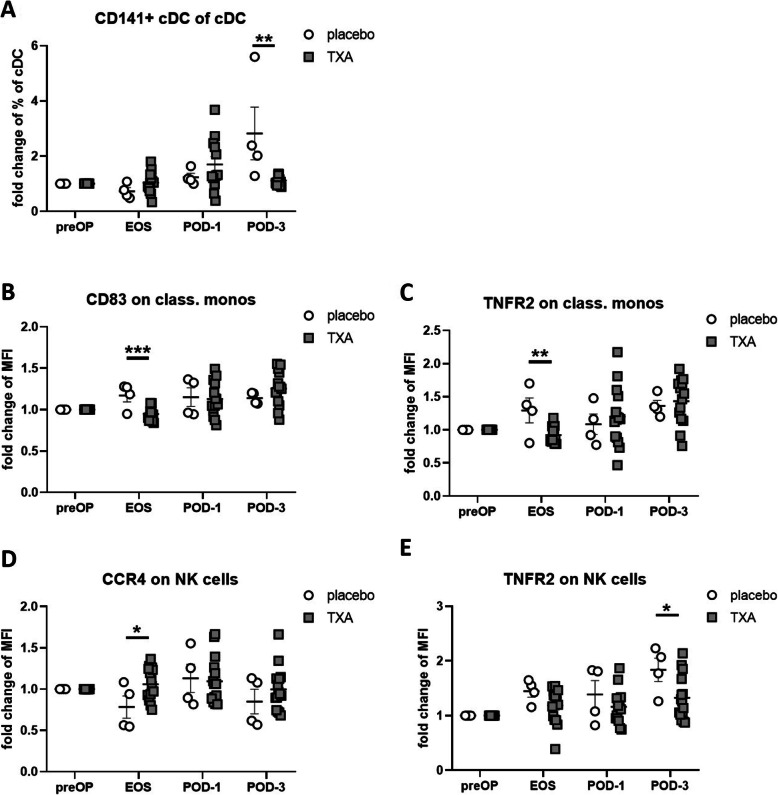
Effects of TXA on white blood cells in orthopaedic surgery**.** The effects of TXA on the assessed immune parameters in orthopaedic surgery were evaluated and a significant reduction in the proportion of CD141+ cDC within overall cDC cells at the POD-3 time point (**A**) was observed. Moreover, a significant decrease in the expression of CD83 (**B**) and TNFR 2 (**C**) on classical monocytes at the EOS time point was detected. In addition, an increase in CCR4 expression on NK cells at EOS (**D**) and a reduction of TNFR2 at POD-3 (**E**) was noticeable in TXA-treated patients. Data represent fold-change from preOP levels and are expressed as mean ± standard error of the mean. Placebo: n = 4, TXA n = 14–15. *p = 0.05, **p = 0.01, ***p = 0.001, 2-tailed Student t test. preOP: pre-operative, EOS: end of surgery, POD-1: post-operative day 1, POD-3: post-operative day 3

We then evaluated the effects of TXA on the assessed immune and haemostasis parameters in orthopaedic surgery in comparison with placebo-treated patients, and observed a significant reduction in the proportion of CD141+ cDC within overall cDC cells at the POD-3 time point (Fig. 6A).

With respect to the expression of surface markers, we detected a significant decrease in the expression of CD83 (Fig. 6B) and TNFR 2 (Fig. 6C) on classical monocytes at the EOS time point. (Fig. 6D)*.* Moreover, we observed an increase in CCR4 expression at EOS (Fig. 6E) as well as reduced expression of TNFR2 on NK cells at POD-3 in TXA-treated patients.

**Fig. 7:**
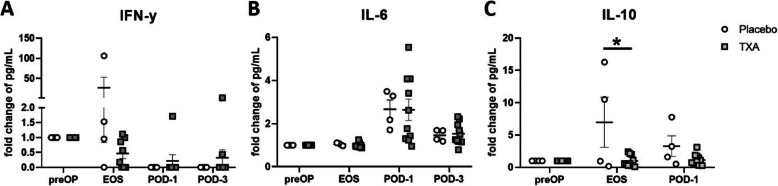
Effects of TXA on T cell cytokines in orthopaedic surgery. Levels of central T cell cytokines were evaluated with ELISA to better characterise the effects of TXA on the T cell response. The Th1 cytokine IFN-y (**A**) and the proinflammatory mediator IL-6 (**B**) were not significantly changed by TXA, while the surgery induced increase in immunosuppressive IL-10 was dampened by TXA at the EOS time point (**C**). Data represent fold-change from preOP levels and are expressed as mean ± standard error of the mean. Placebo: n = 4, TXA: *n* = 12–13. *p = 0.05, 2-tailed Student t test. preOP: pre-operative, EOS: end of surgery, POD-1: post-operative day 1, POD-3: post-operative day 3

To further evaluate the functional consequences of these changes in cell surface marker expression particularly on the T cell response, we determined the plasma levels of the central T cell cytokines IFN-y, IL-6, and IL-10 (Fig. 7). While IFN-y (Fig. 7A) and IL-6 (Fig. 7B) were not significantly altered by TXA, IL-10 showed a significantly suppressed increase at EOS in TXA-treated patients, compared with placebo-treated individuals (Fig. 7C). Due to limited capacity with respect to the ELISA kit, the POD-3 time point was not assessed in this assay.

**Fig. 8:**
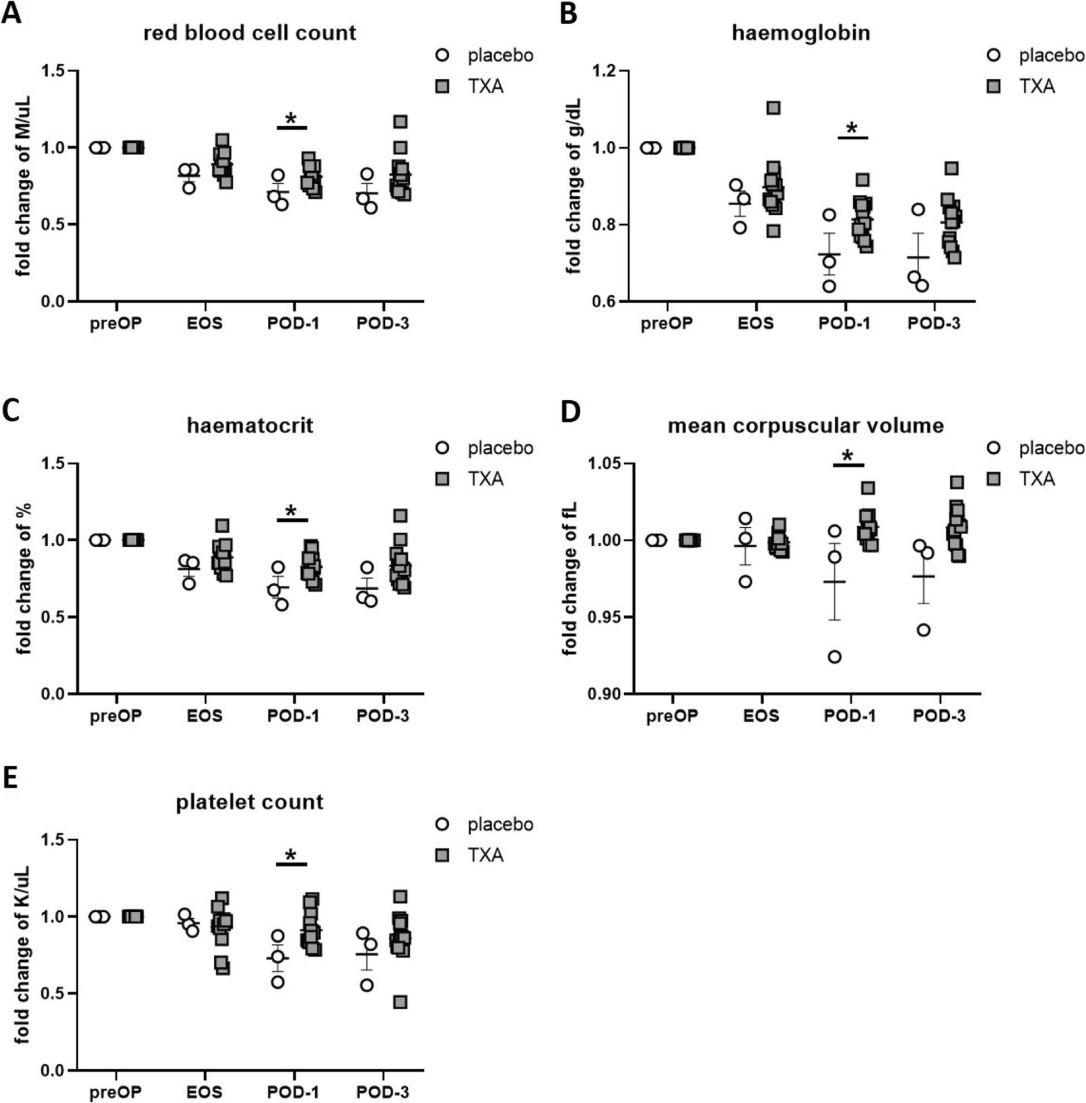
Effects of TXA on red blood cells and platelets in orthopaedic surgery. The effects of TXA on red blood cell parameters as well as platelet counts were assessed, and a significantly reduced decrease in red blood cell count (**A**), haemoglobin levels (**B**) and haematocrit (**C**) at POD-1 could be observed. The mean corpuscular volume of red blood cells was also significantly increased at POD-1 in TXA-treated patients (**D**). A reduced decrease was also evident for platelet count at POD-1 in the TXA group (**E**). Data represent fold-change from preOP levels and are expressed as mean ± standard error of the mean. Placebo: n = 3, TXA: *n* = 15. *p = 0.05, 2-tailed Student t test. preOP: pre-operative, EOS: end of surgery, POD-1: post-operative day 1, POD-3: post-operative day 3

We next assessed the effects of TXA on RBC as well as platelet counts as compared with placebo-treated patients. We observed a significantly reduced surgery-induced decrease in RBC count (Fig. 8A), haemoglobin levels (Fig. 8B) and haematocrit (Fig. 8C) at POD-1. The mean corpuscular volume of RBC was also significantly increased at POD-1 in TXA-treated patients (Fig. 8D). A reduced decrease was also evident for platelet count at POD-1 in the TXA group (Fig. 8E).

Surgery resulted in an elevation of circulating PAP levels. However, TXA did not attenuate the surgery-induced increase (Suppl. Fig. 5).

## Discussion

We have recently demonstrated, that in cardiac surgery patients enrolled into the “Aspirin and Tranexamic Acid for Coronary Artery Surgery” (ATACAS) trial, TXA treatment resulted in enhanced immune activation and a reduction of overall infection rates in patients without diabetes [3]. Similar changes in the immunophenotype of assessed leukocyte subsets could also be observed in healthy volunteers taking 1 g of TXA orally in the same study [3].

In this investigation we were aiming to verify the observations made in cardiac surgery and healthy volunteers also in orthopaedic surgery, in which TXA is frequently used empirically as an antifibrinolytic to reduce blood loss [8]. While meta-analyses have already indicated efficacy and safety of TXA in orthopaedic trauma surgery [9–11], controversy about its use in non-cardiac surgery remains [5]. Data from randomised trials in orthopaedic surgery have so far been scarce, which is why the TALLAS trial was initiated. We recruited patients enrolled into the TALLAS feasibility study [4], and obtained blood samples at the same time points as previously in the ATACAS trial, being preOP, POD-1 and POD-3. In addition, however, by isolating PBMCs for later batch analysis rather than analysing RBC-lysed whole blood samples immediately after collection, we were able to include the EOS time point in the characterisation of the cellular immune response as well.

Furthermore, in this analysis we have successfully implemented the detection of B cells through negative gating as previously described by Blimkie et al. [7] into our previously established 15-colour flowcytometry panels to identify and describe various innate immune cells and lymphocytes [6]. This also provides an additional aspect to our TALLAS sub-study, as B cells were not assessed in the ATACAS investigation. Another addition to the protocol of our TALLAS sub-study was to evaluate alterations in circulating neutrophils, by using FBE, further enhancing our previous investigations on the immune-modulatory effects of TXA in major surgery.

The decision to enrol more patients after the TALLAS pilot trial was finalised was made to confirm the results in a validation trial, and to increase the statistical power of our investigation, to detect potentially more subtle changes, before any analysis was initiated. No difference between patients in the TXA group recruited during or after the randomisation phase was observed in any of the parameters. Baseline characteristics were similar between the placebo and TXA groups. In our previously published ATACAS sub-study, we found that both the blood-sparing and the immune-modulatory effects of TXA were much more pronounced in patients without diabetes, while patients with diabetes were refractory to TXA effects [3]. In contrast to the ATACAS sub-study, where about one third of enrolled patients had diabetes, none of the 9 randomised patients had diabetes or developed an infection in the post-operative phase. In the additionally recruited 10 patients receiving TXA there was one diabetes patient and another one who developed an infection in the 30-days post-operative phase, which however did not result in noticeable changes in any of the assessed parameters. Hence, due to the low number of participants the effect of TXA on post-operative infection rates could not be conclusively evaluated.

Albeit the main comparison is between placebo and TXA treatment, assessing the 4 patients receiving placebo or TXA longitudinally allowed us to gain insight into the effect of lower limb surgery alone on cellular immune parameters. Since the same biomarkers were analysed, we found that the detected changes were similar to our observations in cardiac surgery where a more substantial placebo group was analysed [3], supporting the robustness of the data in this exploratory cohort. To the best of our knowledge, our investigation provides the most extensive characterisation of the cellular immune response in orthopaedic surgery performed so far, both in placebo- and TXA-treated patients. The alterations induced by surgery are consistent with previously published literature, and are proinflammatory and immunosuppressive in nature [12].

Curiously, we detected a significant decrease in the expression of the activation marker CD83 on classical monocytes at the EOS time point, which is opposite to our observations in cardiac surgery patients, where TXA treatment increased expression of CD83 at the later POD-3 time-point [3]. A significant increase of CD83 on classical monocytes was in fact one of the key findings explaining the reduced infection rates with TXA in cardiac surgery patients without diabetes [3]. Hence, our data suggest, that modulation of CD83 expression on phagocytes is a key target of TXA with respect to its immunomodulatory activity, with temporal variations. A reduction of TNFR2 at EOS, suggesting reduced responsiveness to cytokine signalling was an additional observation we made at this early postoperative time point. Neither neutrophils nor B cells were affected by TXA, which is a key new finding.

TXA-treated patients furthermore displayed enhanced expression of CCR4 on NK cells at EOS, a marker mediating migration towards the site of inflammation [13], which may result in reduced NK cell activity at the surgical site. At POD-3 there was moreover a significant reduction of TNFR2 on NK cells notable. Among its diverse functions, TNFR2 has recently been shown to augment TNF-α induced release of IFN-γ, a central cytokine in T helper-type (Th) 1 immune responses from NK cells [14]. Curiously, we also observed an increase of TNFR2 expression on NK cells compared to placebo at the preOP time point, hence before drug treatment could have had any impact. Only one of the individuals recruited to this TALLAS substudy exhibited diabetes and another one developed an infection in the 30 days postoperative phase, yet these important aspects do not explain this baseline difference. Furthermore, no differences between the placebo group and the TXA group were noted with respect to sex, age and type of orthopaedic surgery. While the exact cause for this observation remains unclear, it has to be highlighted here, that comparisons between placebo and TXA groups at the various time points were performed only after normalising the data by presenting them as a fold change from baseline (preOP), specifically to account for any (even non-significant) baseline differences that might trigger a notable difference at later time points not actually induced by TXA treatment itself.

In an attempt to evaluate the functional consequences of these changes in cell surface marker expression particularly on the T cell response, we determined the levels of central T cell cytokines, such as IFN-y, a Th-1 cytokine, IL-10, a Th-2 cytokine, known to be immunosuppressive in the setting of surgery-induced infections, and IL-6, a proinflammatory mediator [12]. In contrast to the recent investigation by Grant et al., showing a TXA-mediated increase in the proinflammatory cytokines MCP-1, TNF-α, IL-1β and IL-6 after orthopaedic surgery [15], we did not observe an increase in IL-6, yet a reduction of the immunosuppressive cytokine IL-10 associated with TXA. Differences in the two investigations have to be acknowledged here, such as in the study design (randomisation vs. non-randomised TXA use [15]), surgery modalities (total hip replacement and total knee replacement vs. total knee replacement only [15]), dose regimen (15 mg/kg and rate of 50 mg/min intravenously at skin incision (for total hip replacement) or just prior to tourniquet release (for total knee replacement) and repeated at 8 and 16 h post-operatively vs. TXA before (IV, 1.2 g/90 kg) and immediately after surgery (intra-articular, 1.4 g/90 kg) [15]). Notably, we observed a wide range in detected cytokine levels particularly at the EOS time point for IFN-y and IL-10, and at POD-1 for IL-6, suggesting profound differences in the individual cytokine response to orthopaedic surgery. At the other postoperative time points, results appeared to be more homogenous across analysed patients.

In this orthopaedic patient cohort, we could confirm that TXA as an antifibrinolytic agent effectively reduced the surgery-induced decrease in RBC count, haemoglobin levels and haematocrit as well as platelets at POD-1, representing its blood-sparing effects. Notably, while the blood-sparing effects of TXA were most evident at the POD-1 time point, we could detect novel cellular immune-modulatory effects of TXA already at the EOS time point. The effects of TXA explaining the immune-stimulating effect of TXA in cardiac surgery [3], such as an increase in CD83 expression on classical monocytes in the post-operative days, were not entirely replicated in this investigation. In fact, the attenuated CD83 expression on classical monocytes at EOS indicates, that TXA may exert opposite effects on the same phagocytic cell subset in a time-dependent manner.

It has to be emphasised here again, that TXA exerts pleiotropic effects on cellular immune function, some of which are mediated through a direct effect, namely inhibited lysine-dependent interaction of plasmin(ogen) with plasminogen receptors on the cell surface of antigen-presenting cells, such as monocytes and DC [16] or the activation/inhibition of soluble inflammatory mediators [1]. It has been demonstrated to activate the proinflammatory mediators IL-8 and MCP-1, as well as to activate the anaphylatoxins C3a and C5a and matrix metalloproteinases, which are crucial in inflammatory cell migration through the extracellular matrix [1]. In contrast, plasmin has also been reported to stimulate expression of the anti-inflammatory and immunosuppressive cytokine TGF-ß [17]. Hence, plasmin(ogen) itself is known as a proinflammatory mediator [1], yet studies have also demonstrated its contribution to the resolution of inflammation [18, 19], which may also partly explain the time-dependent opposing effects of TXA, as an inhibitor of lysine-dependent plasmin generation [8], on phagocyte immunophenotypes. An indirect effect can moreover be expected simply through the reduced blood loss with TXA during surgery, which has been confirmed also in this study. Moreover, it has to be considered that TXA, a lysine derivate [8], would potentially inhibit the interaction of lysine residues with any other binding partner as well, irrespective of the plasminogen activation system. Lysine binding sites have been implicated in protein structures and crosslinking of connective tissue [20], epigenetics [21], and fatty acid metabolism [22]. Hence, TXA may interfere also with these processes, which might be involved in postoperative wound healing and local inflammation.

In our previous studies on TXA in cardiac surgery we found that TXA completely blocked fibrinolysis, yet paradoxically increased PAP levels, representing plasmin formation, at the EOS time point [23]. To mechanistically explore whether the increase in PAP levels in cardiac surgery may be a result of the exposure of blood to negatively charged plastic surfaces in the cardiopulmonary bypass, we also assessed PAP levels in this cohort of orthopaedic surgery patients recruited to the TALLAS pilot trial, also randomised to TXA. While we did not observe a TXA-induced elevation of PAP levels at EOS, TXA clearly did not attenuate the surgery-induced increase, confirming that plasmin generation occurs in the presence of TXA in both cardiac and orthopaedic surgery patients. Therefore, cardiopulmonary bypass-induced contact activation is unlikely the mechanism that gets promoted by TXA and explains the increase in PAP levels at EOS in cardiac surgery. TXA has the potential to enhance plasmin generation by inducing a conformational change of the plasminogen molecule through binding of TXA, which makes it more prone to become activated by plasminogen activators, both by tissue-type plasminogen activator [24] and urokinase [25]. Hence, while plasmin generation on the fibrin surface and other sources of lysine residues is inhibited by TXA, the formation of plasmin, and subsequently PAP in the solution phase (i.e. in plasma) may in fact be augmented by TXA. This also has a potential impact on the inflammatory response to surgery, given the previously mentioned activation of proinflammatory mediators and complement components in the fluid phase, yet we did not observe any difference in proinflammatory cytokine activation in our analysis.

## Conclusions

In conclusion, we extended our examination around the immunological implications of TXA treatment in major surgery by also characterising the EOS time point and including B cells and neutrophils in our immune analysis. As early as at EOS already, TXA administration results in immune-modulation. In addition, we report on TXA-induced changes in RBC and platelet levels at the assessed time points after orthopaedic surgery, as well as a lack of reduction in plasmin formation with TXA. This investigation therefore enhances our understanding of TXA-mediated effects on the haemostatic and immune response in major surgery.

## Supplementary Information

**Additional file 1: Supplementary Figure 1.** Gating strategy used to identify myeloid cells. **Supplementary Figure 2.** Gating strategy used to identify B cells. **Supplementary Figure 3.** Gating strategy used to identify NK cells. **Supplementary Figure 4.** Comparison of results for TXA-treated patients in the randomised and after the randomised recruitment. **Supplementary Figure 5.** Plasmin-antiplasmin (PAP) complex as a readout for plasmin generation.

## Data Availability

All data relevant to this manuscript are published directly in the main manuscript or the supplementary material.

## Abbreviations

cDC: Conventional dendritic cells
EOS: End of surgery
FBE: Full blood examination
MFI: Mean fluorescence intensity
MO-MDSC: Monocytic myeloid-derived suppressor cells
PBMCs: Peripheral blood mononuclear cells
pDC: Plasmacytoid dendritic cells
POD-1/3: Post-operative day 1/3
preOP: Pre-operative
RBC: Red blood cells
SSC: Side scatter
Th-1/2: T helper-type 1/2
TXA: Tranexamic acid
WBC: White blood cells
