# Supplementary material for: Tranexamic acid alters the immunophenotype of phagocytes after lower limb surgery
Source: Thromb J. 2022 Apr 11;20:17. doi: 10.1186/s12959-022-00373-3 (PMC8996554; doi:10.1186/s12959-022-00373-3)
Supplement: Supplementary file 1 — Additional file 1: Supplementary Figure 1. Gating strategy used to identify myeloid cells. Supplementary Figure 2. Gating strategy used to identify B cells. Supplementary Figure 3. Gating strategy used to identify NK cells. Supplementary Figure 4. Comparison of results for TXA-treated patients in the randomised and after the randomised recruitment. Supplementary Figure 5. Plasmin-antiplasmin (PAP) complex as a readout for plasmin generation. [file 12959_2022_373_MOESM1_ESM.docx]

**Supplementary material related to Draxler et al. “Tranexamic acid alters the immunophenotype of phagocytes after lower limb surgery”**

**
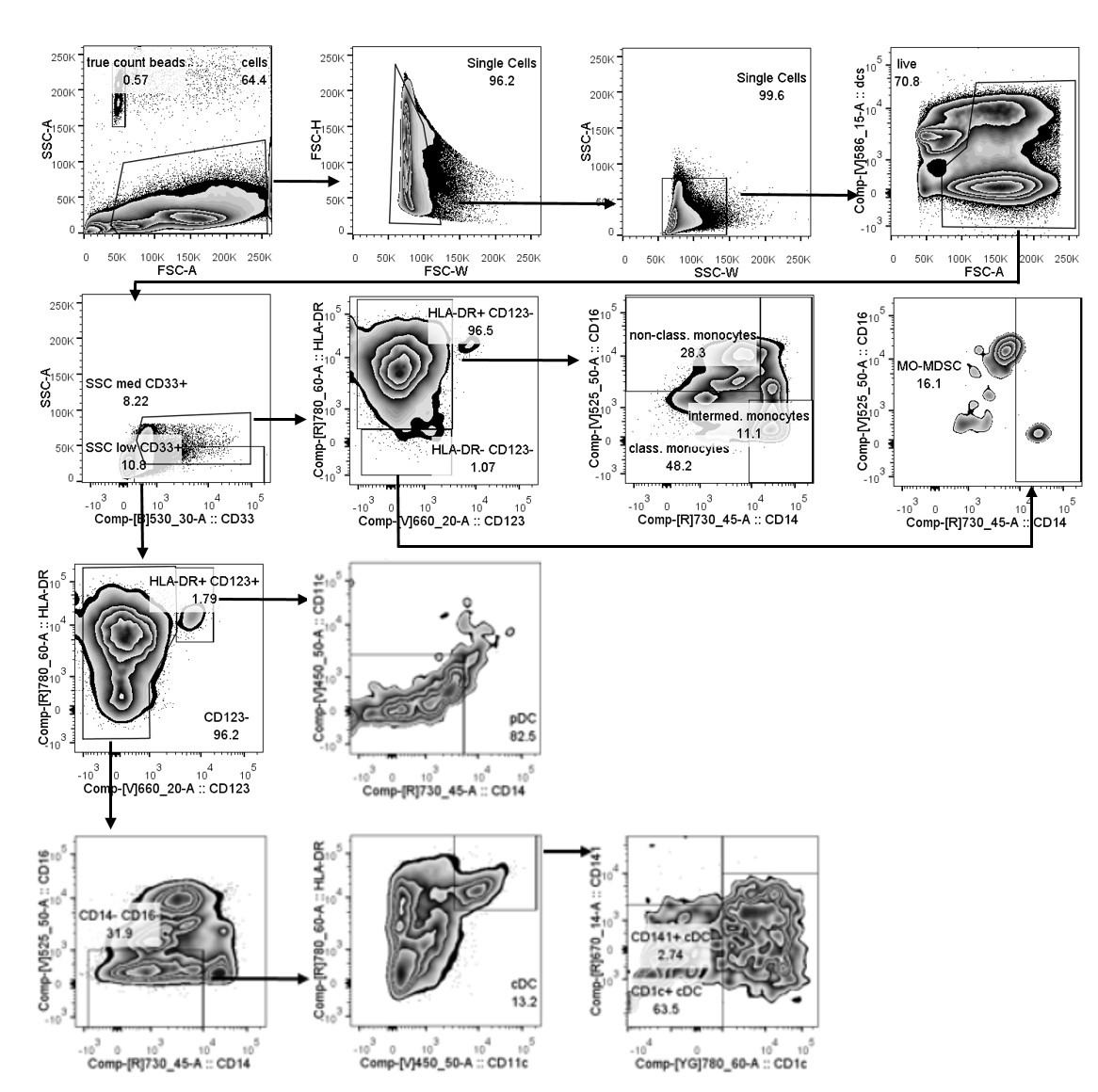
**

**Suppl. Fig.1 Gating strategy used to identify myeloid cells**

After identifying single cells and removing dead cells from the analysed cell population, side scatter (SSC) med CD33+ cells were gated to further distinguish between HLA-DR+ CD123- monocytes and HLA-DR- CD123- MO-MDSC. Monocytes were further divided into the 3 subsets CD14+ CD16- classical, CD14+ CD16+ intermediate, and CD14- CD16+ non-classical monocytes. Within the SSC low CD33+ population, cDC were identified as CD123- CD14- CD16- HLA-DR and CD11c+ cells, and further divided into CD141+ cDC and CD11c+ cDC. pDC were identified as HLA-DR+ CD123+ cells negative for CD14 and CD11c [1].

**
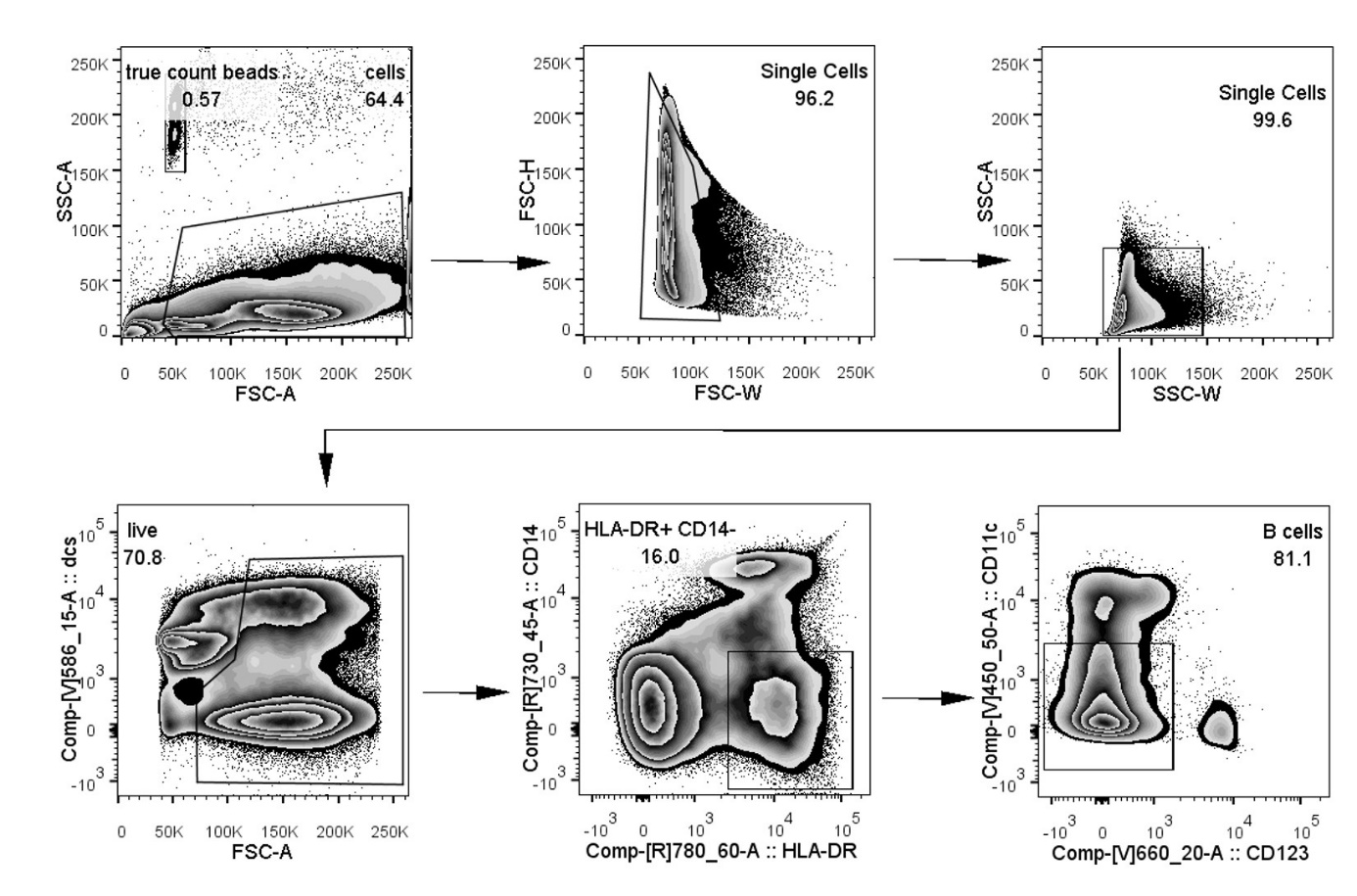
****Suppl. Fig.2 Gating strategy used to identify B cells**

B cells were identified through negative gating as HLA-DR+ CD14- CD11c- CD123- cells, as previously described [2].


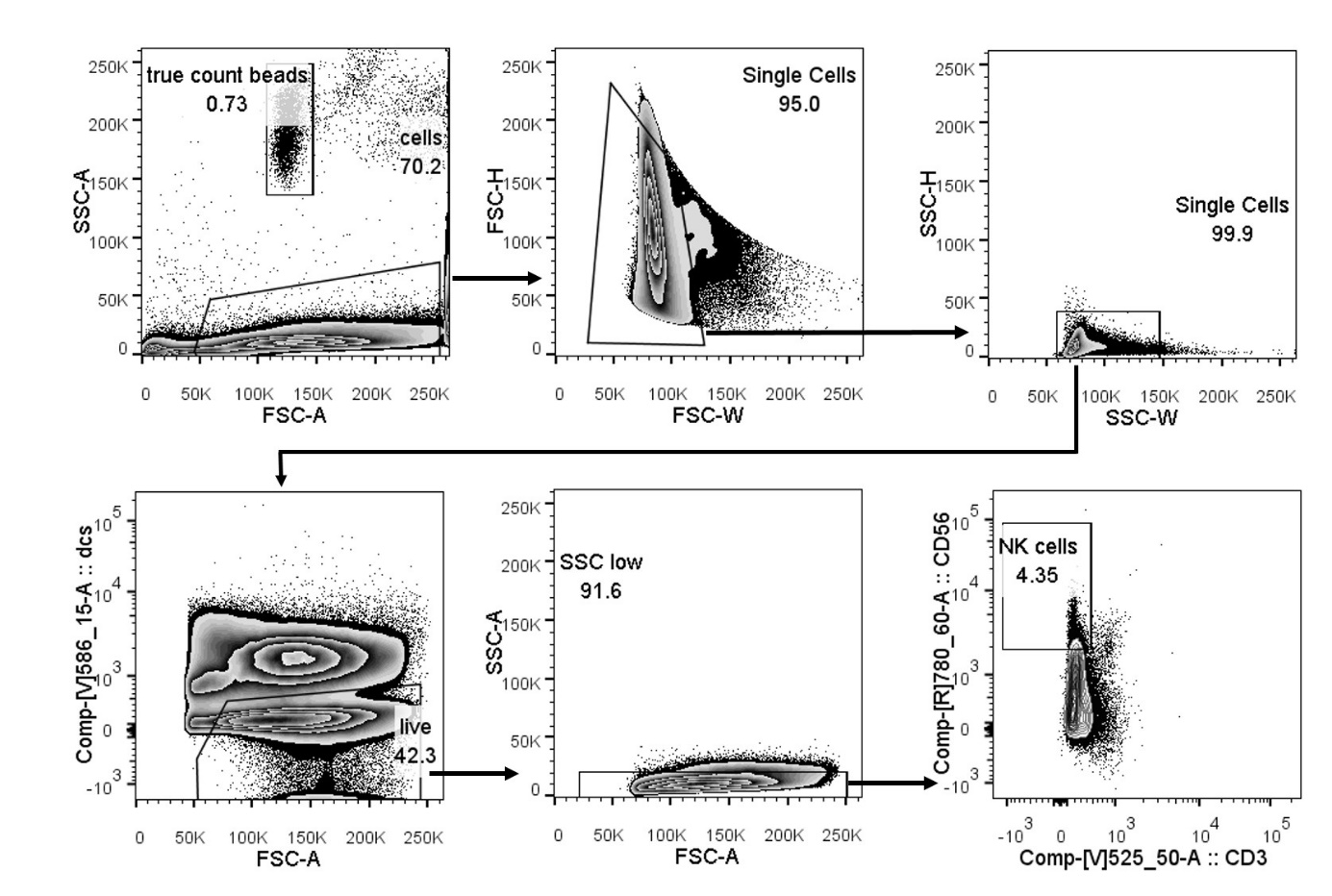


**Suppl. Fig.3 Gating strategy used to identify NK cells**

After identifying single cells and removing dead cells from the analysis, NK cells were identified as side scatter (SSC) low CD3- CD56+ cells [1].

**
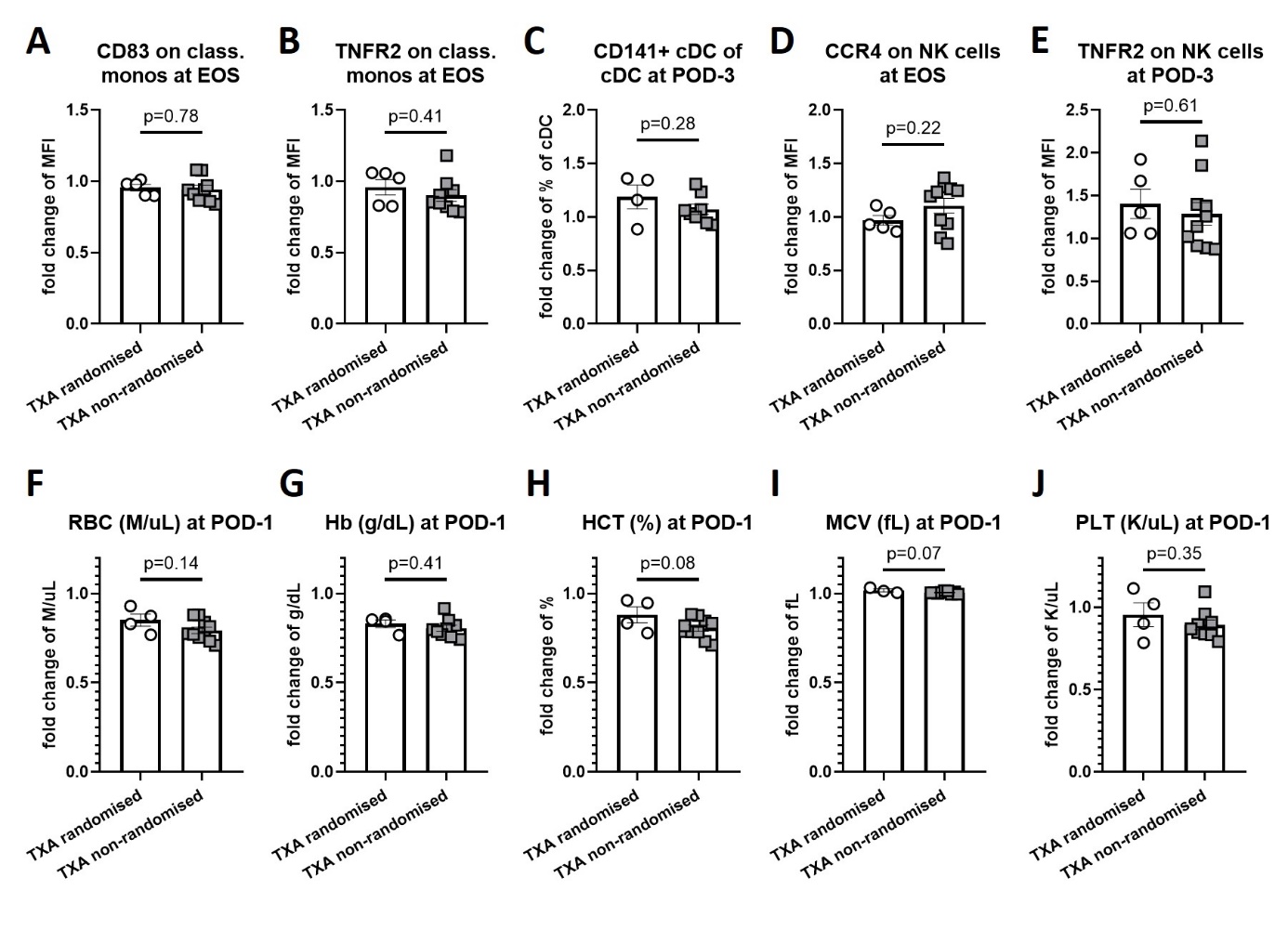
**

**Suppl. Fig.4 Comparison of results for TXA-treated patients before in the randomised and after the randomised recruitment**

For analyses of the 10 additional patients recruited after the randomization phase was finalised, we compared results obtained during the randomization period with results obtained after randomization, which were later pooled for the comparison with the placebo group. No differences were observed between those two TXA groups for CD83 **(A)** and TNFR2 **(B)** expression on classical monocytes at EOS, the proportion of CD141+ cDC within cDC cells at POD-3 **(C)** or the expression strength of CCR4 **(D)** and TNFR2 **(E)** on NK cells at EOS and POD-3, respectively.

Furthermore, RBC count **(F)**, haemoglobin **(G)** and haematocrit **(H)** levels, as well as mean corpuscular volume **(I)** and platelet count **(J)** at POD-1 were not significantly different between TXA-treated patients recruited during or after the randomization phase.

Error bars represent mean ± standard error of the mean. “TXA randomised”: n=4-5, “TXA non-randomised”: n=9-10. Analysis was performed using an unpaired Student’s t test, EOS: end of surgery, POD-1: post-operative day 1, POD-3: post-operative day 3

**
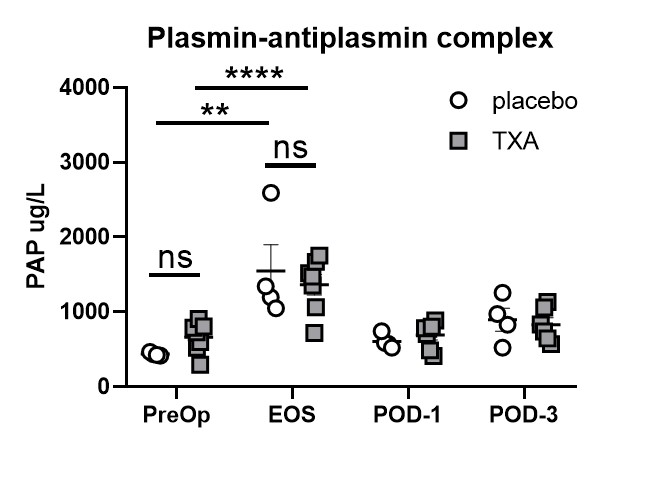
**

**Suppl. Fig.5 Plasmin-antiplasmin (PAP) complex as a readout for plasmin generation**

Surgery induced a significant increase in PAP levels, indicating increased plasmin formation at EOS, with no inhibitory effect of TXA.

Error bars represent mean ± standard error of the mean. Placebo: n=4, TXA: n=7. Changes over time were compared with preOP levels with a repeated measures one-way ANOVA with Dunnett’s correction test for multiple comparisons **p=0.01, ****p=0.0001, Comparisons between placebo and TXA at individual time points were performed with 2-tailed Student t test. ns: not significant, preOP: pre-operative, EOS: end of surgery, POD-1: post-operative day 1, POD-3: post-operative day 3

**References**

1. Draxler, D.F., et al., *A flowcytometric analysis to efficiently quantify multiple innate immune cells and T Cell subsets in human blood.* Cytometry A, 2017. **91**(4): p. 336-350.

2. Blimkie, D., et al., *Identification of B cells through negative gating-An example of the MIFlowCyt standard applied.* Cytometry A, 2010. **77**(6): p. 546-51.
